# Nitric Oxide (NO)-Releasing Macromolecules: Rational Design and Biomedical Applications

**DOI:** 10.3389/fchem.2019.00530

**Published:** 2019-07-26

**Authors:** Jian Cheng, Kewu He, Zhiqiang Shen, Guoying Zhang, Yongqiang Yu, Jinming Hu

**Affiliations:** ^1^CAS Key Laboratory of Soft Matter Chemistry, Hefei National Laboratory for Physical Science at the Microscale, Department of Polymer Science and Engineering, University of Science and Technology of China, Hefei, China; ^2^Department of Radiology, The First Affiliated Hospital of Anhui Medical University, Hefei, China

**Keywords:** nitric oxide, antibacterial, anticancer, wound healing, *N*-diazeniumdiolates, *S*-nitrosothiols

## Abstract

Nitric oxide (NO) has been recognized as a ubiquitous gaseous transmitter and the therapeutic potential has nowadays received increasing interest. However, NO cannot be easily directly administered due to its high reactivity in air and high concentration-dependent physiological roles. As such, a plethora of NO donors have been developed that can reversibly store and release NO under specific conditions. To enhance the stability and modulate the NO release profiles, small molecule-based NO donors were covalently linked to polymeric scaffolds, rendering them with multifunctional integration, prolonged release durations, and optimized therapeutic outcomes. In this minireview, we highlight the recent achievements of NO-releasing macromolecules in terms of chemical design and biomedical applications. We hope that more efforts could be devoted to this emerging yet promising field.

## Introduction

Nitric oxide (NO), a gaseous diatom radical, is highly reactive and can be easily oxidized in air, which used to be known as one of the most notorious resources of air pollution. The situation remains until NO was identified as the endogenous gaseous transmitter in the 1980s. NO is continuously produced by the NO synthase-mediated transformation of *L*-arginine into *L*-citrulline. The biomedical functions of NO are highly concentration-dependent (Carpenter and Schoenfisch, 2012). Because it is inconvenient to directly use NO gas in clinical trials, a number of NO donors that can store and release NO have been developed including metal nitrosyl, organic nitrates, *N*-diazeniumdiolates (NONOates), *S*-nitrosothiols (SNO) and so on (Quinn et al., 2015). Indeed, some of them are clinically used for the treatment of diseases such as sodium nitroprusside and nitroglycerin (Nichols et al., 2012). In the past 20 years, it was of increasing interest to unravel the biological functions of NO and to develop novel NO donors. To date, it is known that NO exerts crucial effects in cancer therapy, antibacterial infections, wound healing, host defense and immune response and so on.

**Figure 1:**
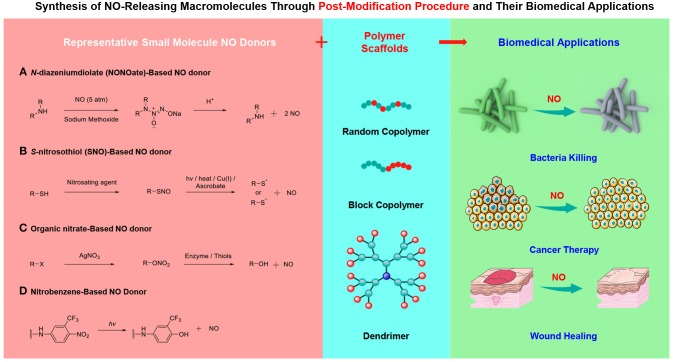
Fabrication of macromolecular NO donors through the incorporation of small molecule NO donors into polymer scaffolds and their biomedical applications.

In this minireview article, we manage to outline the recent achievements of macromolecular NO donors and special attention is paid to the chemical design strategies. Although it was not difficult to appreciate that the fabrication of macromolecular NO donors primarily inherits the design concepts of small molecule NO releasers, the incorporation of small molecule NO donors into polymeric scaffolds results in the formation of polymeric NO donors can not only improve the storage and release performance of NO but also optimize the pharmacokinetics. The following sections are categorized into three parts according to the chemical structures of NO-releasing moieties, which encompasses *N*-diazeniumdiolates (NONOates), *S*-nitrosothiols (SNO), and other NO donors (Figure 1). Finally, a conclusion and outlook section is given. The intention is not to provide an exhaustive literature survey but to showcase many possibilities to construct macromolecular NO donors. The rational design and functional applications of small molecule-based NO donors are now available (Zhou et al., 2016), which are not included in this minireview. Also, the direct mixing small molecule NO donors and polymeric matrices with the formation of NO-doping polymers through non-covalent interactions does not fall within the scope either (Mowery et al., 2000). Without a doubt, this emerging field contains numerous possibilities and the further development of macromolecular NO donors will be further advanced by the cooperation of polymer chemists, materials scientists, biologists, and so on.

## *N*-diazeniumdiolate (NONOate)-Based Polymeric NO Donors

*N*-diazeniumdiolate (NONOate) derivatives are the most widely investigated NO donors and some of them such as diethylamine/NO adduct (DEA NONOate) and spermine NONOate are commercially available. The excellent contributions of Drago and coworkers (Drago and Paulik, 1960; Drago and Karstetter, 1961) made it possible to prepare and purify NONOate derivatives, which are now of extensive use in investigating the physiological function of NO. These compounds are typically synthesized by the reaction of secondary amines with NO under high pressure (e.g., 5 atm) and alkaline condition (e.g., sodium methoxide). NONOate derivatives are relatively stable at basic pH but they spontaneously release 2 moles of NO per mole of donors in the presence of protons. The NO release profiles follow first-order kinetics and the half-lives of NONOates can span from several seconds to hours depending on the chemical structures of the secondary amines (Miller and Megson, 2007). Interestingly, after NO release, NONOate derivatives were transformed into the original amines without the generation of other metabolites. To minimize the uncontrolled release and optimize the pharmacokinetics of NONOate-based small molecule donors, researchers have functionalized amine-containing polymeric matrices to obtain NONOate-based macromolecular NO donors (Sadrearhami et al., 2018). In the seminal contributions, NONOate-based macromolecular NO donors with varying chain topologies were prepared (Smith et al., 1996; Pulfer et al., 1997). They ingeniously demonstrated that the NO-releasing kinetics could be remarkably changed, highly dependent on the spatial location and localized microenvironment of the NONOate moieties.

Inspired by these seminal works, polymeric scaffolds containing amine residues such as hyperbranched poly(ethylene imine) (PEI) have been widely used for the fabrication of macromolecular NO donors (Kang et al., 2015). In a recent study, propylene oxide was used to functionalized with hyperbranched PEI with the formation of hydroxyl moieties and the residual amine groups was treated with NO. *In vivo* study on mice revealed that the resulting macromolecular NO donors exhibited accelerated wound healing performance in full thickness excisional cutaneous wound model (Zhang et al., 2019). The PEI-derived NONOate-based NO donors can also be used as the stabilizing agents of inorganic nanoparticles. As a result, hybrid nanomaterials could be achieved by taking advantages of the physiochemical properties of inorganic nanoparticles and the therapeutic effect of NO (Yu et al., 2018). In this context, silica nanoparticles were coated with PEI and then treated the surface-bound PEI with NO with the formation of NONOate residues (Jeong et al., 2018). These hybrid nanoparticles displayed sustained and prolonged NO release behavior that can be used to treat bacterial keratitis.

The selective reaction between amine residues and NO renders it possible to develop versatile macromolecular NO donors. Besides PEI, methacrylate monomers consisting of linear and cyclic pendant secondary amines in the side chains which were initially protected by *tert*-butyloxycarbonyl (Boc) group were devised and synthesized (Parzuchowski et al., 2002; Zhou and Meyerhoff, 2005). After polymerization and deprotection protocols, the deprotected amine residues were further decorated with NO, generating NO-releasing polymers. In addition to linear chain polymers, cross-linked polymers can readily be synthesized by prior cross-linking of the amine-containing linear precursors. This design concept was further expanded and NONOate-containing block copolymers were synthesized, which self-assembled into micellar nanoparticles in aqueous solution (Jo et al., 2009). Note that the formation of nanoassemblies can not only shield the labile NONOate moieties from protons in water that led to burst NO release but also render the localized delivery of NO possible by taking advantage of enhanced permeability and retention (EPR) effect of micellar nanoparticles.

The Schoenfisch group (Stasko and Schoenfisch, 2006; Lu et al., 2011, 2013; Sun et al., 2012; Worley et al., 2014, 2015) has made tremendous achievements on the development of NO-releasing dendrimers. In an early example, they systematically investigated the effects of the dendritic generations and amine types on the storage and release of NO. Thorough investigations demonstrated that, as compared to the small molecule counterparts, the secondary amine-containing dendrimers showed a unique dendritic effect and exhibited a significantly longer NO release period (Stasko and Schoenfisch, 2006). After elucidating the correlations between the chemical structures and NO-releasing performance, they examined the antibacterial effect of these NO-releasing dendrimers. Although these dendrimers cannot efficiently penetrate bacterial biofilms, the incorporation of NO can drastically boost the anti-biofilm activity (Sun et al., 2012; Lu et al., 2013; Worley et al., 2014, 2015).

Notably, one of the most important goals is to explore the potentials of NO-releasing macromolecules in biological systems. Thus, the biocompatibility of polymer scaffolds is of crucial importance. In this regard, biocompatible polysaccharides (e.g., chitosan and dextran) and oligosaccharides (e.g., cyclodextrin) were also employed as the polymeric matrices to fabricate NONOate-based polymeric NO donors. For example, the amine residues of oligochitosan were first modified with 2-methyl aziridine through ring-opening reaction and the newly formed amine groups were further treated with NO gas (Lu et al., 2014). The NO loading capability, maximum NO flux, and half-lives of the resulting NO-releasing oligochitosan were highly dependent on the molar ratio of 2-methyl aziridine to the amine residues. Cell viability studies revealed that the NO-releasing oligochitosan had minimal toxicity to normal L929 mouse fibroblast but could efficiently eradicate bacterial biofilm. Detailed antibacterial studies revealed that the water solubility, appropriate molecular weights, and ionic characteristics of the NO-releasing chitosan synergistically contributed to the biofilm killing. Recently, mono-substituted and multi-substituted β-cyclodextrin (β-CD) was engineered as NO-releasing materials as well, exhibiting cooperative antibacterial activity by means of NO and antibiotics loaded within the cavity of β-CD via host-guest interaction (Jin et al., 2018).

Although many of the NONOate-based polymeric NO donors were obtained by the functionalization of secondary amine-containing polymers, aliphatic primary amines can also be transformed into NONOate derivatives as well. Recently, a statistical ternary copolymer containing primary amine residues was synthesized (Namivandi-Zangeneh et al., 2018). The primary amine moieties were converted into NONOates in the presence of NO gas under high pressure. The resulting polymers exerted synergistic antibacterial effects by taking advantages of NO-mediated eradication of biofilm and cationic polymer-assisted membrane disruption of bacteria.

As mentioned above, although amine-containing polymers can be functionalized with NO gas to form NONOate-based macromolecular NO donors and the half-lives and pharmacokinetics could be effectively altered, the spontaneous NO release from these NONOate-based polymeric NO donors cannot be eliminated. It will be more promising to develop polymeric NO donors with on-demand release behavior that could avoid premature NO leakage. In this context, the terminal oxygens of NONOate derivatives were protected by glycosidase-responsive galactose moieties (Zhao et al., 2013). As a result, the spontaneous NO release was remarkably inhibited and controlled NO release could be achieved by incubating the NO donors with glycosidase. After attaching the enzyme-responsive NO donors to chitosan backbones through copper(I)-catalyzed azide-alkyne cycloaddition (CuAAC) reaction, the resulting macromolecular NO donors inherited glycosidase-enzyme characteristics. This newly designed NO-releasing material cannot only inhibit platelet adhesion and prolong partial thromboplastin time but also show increased angiogenesis in a diabetic mouse model. Indeed, beside glycosidase, NONOate derivatives can be selectively caged by many other functional groups and selective uncaging reactions could be actuated by light irradiation, glutathione (GSH), and other enzymes such as esterase, nitroreductase, and DT-diaphorase (Makings and Tsien, 1994; Sharma and Chakrapani, 2014). The introduction of protected group chemistry opens a new avenue to devise stable NONOate donors, which should be more advantageous in biomedical application due to the possibility to minimize premature NO release and accomplish on-demand NO release at regions of interest.

## *S*-nitrosothiol (SNO)-Based NO Polymeric NO Donors

In comparison with exogenous NONOate-based donors, SNOs have been recognized as endogenous transports of NO and *S*-nitrosoglutathione (GSNO) and *S*-nitrosocysteine (CysNO) have been identified in biological systems. SNO derivatives were generally synthesized in aqueous solution by the modification of thiols in the presence of nitrosating agents such as nitrogen dioxide (NO_2_), dinitrogen tetroxide (N_2_O_4_), dinitrogen trioxide (N_2_O_3_), and nitrite (${\text{NO}}_{2}^{-}$). The nitrosation reactions can also be implemented in an organic solvent using *tert*-butyl nitrite as the nitrosating agent. The bond energy of S-NO was calculated to be ~150 kJ/mol, which was even lower than that of a redox-responsive disulfide bond (~240 kJ/mol) (Fan et al., 2015a). Because of the low bond energy, the NO release from SNO derivatives can be readily activated by light irradiation, heat, metal ions [e.g., Cu(I)], ascorbate and so on.

Akin to that of polymeric NONOate donors derived from amine-containing polymers, macromolecular SNO-based donors could be prepared from polymer precursors having thiol residues (Coneski et al., 2010; Coneski and Schoenfisch, 2011). It is known that reversible addition-fragmentation chain transfer (RAFT) polymerization could be used for the synthesis of polymers with varying compositions and chain topologies. The as-prepared polymers with RAFT agents at the chain terminals could be converted into free thiol groups. As such, it would be straightforward to synthesize SNO-terminated polymers via the combination of RAFT polymerization, RAFT agent removal, and nitrosation modification. For example, the benzodithioate terminal of a diblock copolymer synthesized by RAFT polymerization was successively transformed into a free thiol and SNO motif in the presence of hydrazine and nitrite, respectively (Yu et al., 2015). The NO release kinetics can be modulated by the solution pH and the formation of micellar nanoparticles at basic condition can markedly slow down the NO release rate (Hu et al., 2014a).

As mentioned above, amine-containing polymers can be successfully transformed into NONOates. Notably, they can also be engineered as SNO-type NO donors via an *S*-nitroso-*N*-acetylpenicillamine (SNAP) derivation approach. The SNAP approach provides a robust procedure to functionalize both natural and synthetic peptides bearing amine residues (e.g., lysine) to form NO donors. For instance, a natural protein, fibrin, was decorated with SNAP with the formation of NO-releasing peptide and the resulting peptide can remarkably inhibit bacterial adhesion compared with natural fibrin without NO-releasing capability (Vanwagner et al., 2013). Besides peptides, natural polysaccharides and oligosaccharides could also be modified with the formation of SNO-type NO donors. For example, the amine residues of chitosan can be easily transformed into thiols with Traut's reagent (i.e., 2-iminothiolane hydrochloride); the resulting thiols underwent nitrosation reaction with the formation of SNO-containing macromolecular NO donors. The NO release could be activated by endogenous ascorbic acid, eliciting a 4-log reduction in the viability of *Pseudomonas aeruginosa* (Lu et al., 2015). As a representative example of oligosaccharide, β-CD has been extensively investigated in host-guest chemistry (Hu and Liu, 2014). Because the hydroxyl groups in β-CD could be selectively functionalized, β-CD with one and seven SNO moieties were synthesized (Piras et al., 2013). Moreover, the mono-substituted β-CD-SNO can still be used as a host molecule to include specific guest molecules such as tamoxifen citrate and *N*-desmethyltamoxifen hydrochloride, which may show synergistic therapeutic performance.

## Other Polymeric NO Donors

Apart from NONOates and SNOs, other potential NO donors could be incorporated into polymer scaffolds such as organic nitrates and nitrobenzene derivatives. It is known that NO has a high affinity to many transition metal ions and transition ion-containing molecules can thus be used for NO storage (Wang et al., 2017, 2018). Although metal nitrosyls have been clinically prescribed, the covalent attachment of metal nitrosyls to macromolecular scaffolds has rarely been investigated, possibly due to the difficulties in chemical modification of metal nitrosyls. To obtain organic nitrates, halogenated alkyl precursors were typically treated with silver nitrate (AgNO_3_). Recently, 2-(nitrooxy)acetic acid was conjugated to hyaluronic acid via esterification reaction and photothermal agent (e.g., indocyanine green; ICG) and chemotherapeutic drug (e.g., doxorubicin; DOX) were simultaneously loaded into the nanoparticles. The resulting multifunctional nanoparticles exhibited hyaluronidase (HAase)-mediated size shrinkage and near-infrared (NIR) light-triggered NO release, exerting a synergistic effect on cancer therapy (Hu et al., 2018). Intriguingly, besides NIR light, organic nitrates also responded to endogenous reducing agents such as GSH (Duong et al., 2014).

On the other hand, nitrobenzene derivatives have been developed as photoresponsive NO donors, exhibiting good stability without light irradiation yet photo-triggered NO release upon light exposure. In this aspect, the Sortino group and other researchers have focused on 4-nitro-3-(trifluoromethyl)aniline derivatives and demonstrated these photoresponsive NO-releasing molecules having board biomedical applications (Caruso et al., 2007; Kandoth et al., 2014; Rapozzi et al., 2015; Fraix and Sortino, 2018). Photo-mediated NO release from 4-nitro-3-(trifluoromethyl)aniline was ascribed to the presence of trifluoromethyl group in the ortho position that forced the nitro group in a twisted geometry. Recently, 4-nitro-3-(trifluoromethyl)aniline and lectin-binding _D_-mannopyranoside derivatives were attached to an alternative copolymer of poly(styrene-*alt*-maleic acid) (Yang et al., 2013). Interestingly, the NO release from the resulting polymer can be actuated by chemiluminescence process derived from luminol/horseradish peroxidase (HRP) system, which overcame the drawback of the poor tissue penetration of exogenous light irradiation.

Notably, the introduction of bulky groups to nitrobenzene derivatives in a proximal position renders them responsive to light with the capability of releasing NO, which has proved to be a general and efficient method toward nitrobenzene-based NO donors (Suzuki et al., 2005; Hishikawa et al., 2009; Horinouchi et al., 2011; Nakagawa et al., 2013). To further elevate the photosensitivity and light-triggered NO release performance, *N*-nitrosoamine derivatives were developed, which can be cleaved by UV/visible/NIR light irradiation (Namiki et al., 1997; Ieda et al., 2014; Zhou et al., 2018), ultrasonication (Jin et al., 2017), heat (Fan et al., 2015b) and so on, highly relying on the chemical structures (Ohwada et al., 2001). In addition, furoxan derivatives that can selectively release NO trigged by thiol-containing molecules were also integrated into the polymeric system to fabricate polymeric NO donors (Wang et al., 2015a; Poh et al., 2017). Significantly, compared with labile NONOates and SNOs, furoxan derivatives were compatible with typical CuAAC reaction conditions, providing many possibilities to construct NO-releasing polymers.

## Conclusions and Outlook

In this minireview, we have summarized the recent achievements of polymeric NO-releasing materials in terms of chemical design strategies and biomedical applications. These polymeric NO donors can be roughly divided into three categories according to the chemical structures and display promising applications in anticancer, antibacterial, wound healing and so on (Table 1). Only selective literature reports are discussed, but it is not difficult to observe that this emerging field is now receiving increasing interest, particularly for exploring the biomedical applications of these NO-releasing macromolecules. Despite tremendous achievements, there remain some challenges to be resolved in future studies.

Table 1Summary of the properties of NO donors and working mechanisms in biomedical applications.

**Table:** 

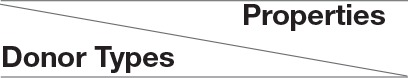		**Releasing Triggers**	**Advantages**	**Drawbacks**
	NONOates	Acidic pH	commercially available; releasing 2 moles of NO per mole of NONOate	Tough synthetic conditions; unstable at physiological conditions
	SNOs	Light, X-ray, heat, reducing agents, Cu(I) and so on	Endogenous NO carrier (good biocompatibility); without tolerance with long-term use	Unstable at physiological conditions
Other Donors	Metal Nitrosyls	Enzymes, Light, reducing agents and so on	Clinically applied	Potential cytotoxicity of heavy metal ions and spontaneous ligand exchange in biological conditions
	Organic nitrate	Enzymes, reducing agents	Clinically applied	Cannot be released without specific enzymes; generation of tolerance
	Nitrobenzene derivatives	Light	Relatively stable in physiological conditions; decreased premature NO leakage	Heavily focused on ultraviolet light
	*N*-nitrosoamine Derivatives	Light, heat, ultrasound and so on	Relatively stable in physiological conditions; decreased premature NO leakage	Heavily focused on ultraviolet light

**Table:** 

**Classification**	**Typical Working Mechanisms**
**BIOMEDICAL APPLICATIONS OF NO-RELEASING MACROMOLECULES**	
Wound Healing	NO participates all the phases of wound healing including vasodilation and antiplatelet effects during the inflammation process, promotion of reepithelialization and angiogenesis during the proliferative phase, and enhanced collagen deposition during the remodeling phase.			
Antibacterial action	NO serves as a major signal for biofilm dispersal at low concentration (e.g., nM) and NO can mediate chemical alternation of DNA and inhibits DNA repair at high concentration (e.g., >μM).			
Cancer Therapy	NO exhibits multifactorial effects in cancers and a high concentration (e.g., > μM) of NO leads to deamination of DNA bases, nitrosylation of enzymes and proteins, cellular dysfunction, elevated inflammatory reactions, and cell apoptosis. NO can also integrate with other therapeutic techniques such as photodynamic therapy, radiotherapy, and chemotherapy to improve therapeutic outcomes.			

First, because the unstable nature of many NO donors such as NONOates and SNOs, the preparation of polymeric NO donors was dominantly achieved through a postmodification approach. Although the stability of NO-releasing precursors could be efficiently elevated and the release durations could be modulated by the polymeric scaffolds, the uncontrolled release nature cannot be eliminated. To minimize the side effects of NO, polymeric NO donors with controlled release performance are more appealing when considered their biomedical applications. To this end, it is of crucial importance to screen novel NO donors with sufficient stability at physiological conditions and triggered NO release under specific stimuli such as non-invasive light and endogenous stimuli (e.g., acidic pH, redox, and overexpressed enzymes). The development of organic nitrate- and nitrobenzene-based NO donors can significantly increase the stability of NO donors, whereas many of the nitrobenzene-based NO donors were only responsive to ultraviolet (UV) light with poor tissue penetration.

Second, the developed polymeric NO donors through the postmodification approach rendered it difficult to tune the self-assembly behavior of polymeric NO donors due to the non-specific modification. To date, the self-assembly behavior of polymeric NO donors was far less explored and only a few examples of micelle-based NO carriers have been reported. Recent results suggested that the NO-releasing moieties could be introduced into the preformed nanostructures by the combination of polymerization-induced self-assembly (PISA) and postmodification approach (Sadrearhami et al., 2017). Indeed, the spatial location of NO donors within the assemblies greatly affected the NO-releasing kinetics. Given the shape-dependent interactions between cells and nanoassemblies, it is expected that other self-assembled morphologies (nanorods, vesicles, etc.) likely altered the extracellular and intracellular NO delivery performance (Hu et al., 2013). Therefore, the self-assembly behavior of polymeric NO donors should be investigated. In this context, the stability of polymeric NO donors should be a prerequisite since the spontaneous NO release will change the chemical compositions and in turn affect the self-assembly behavior. On the other hand, besides the postmodification method, new synthetic strategies such as direct polymerization of NO-releasing monomers appears to be an option, possibly generating well-defined block copolymers capable of self-assembling into various nanostructures (Wang et al., 2015b; Deng et al., 2016, 2018).

Finally, small molecule-based NO donors such as nitroglycerin and sodium nitroprusside have been widely used in clinical trials (Nichols et al., 2012). Preliminary results of polymeric NO donors have revealed promising perspective in biomedical applications. Besides NO itself, it can also be delivered with other therapeutic agents to reverse multidrug resistance and thus improve therapeutic outcomes. However, it appears that the macromolecular NO donors cannot easily bypass the long-standing difficulties of other polymeric nanomedicines including relatively low delivery efficiency, undesirable biodistributions, and systemic side effects. On the other hand, it is well-documented that the biological functions of NO are highly concentration dependent. Besides NO donors that can selectively release NO in the pathological environment, the development of NO-responsive polymers that can efficiently scavenge endogenous NO may open new avenues for specific disease therapy (Hu et al., 2014b, 2015a,b,c, 2016; Liu et al., 2017; Zhang et al., 2017; Ding and Hu, 2018). Although many efforts have to be devoted to resolving the current problems of polymeric NO donors as well as other macromolecular nanomedicines, we believe that the continuous development of nanotechnology, polymer chemistry, and biology will make polymeric NO donors and other nanomedicines smarter that could finally conquer the difficulties.

## Author Contributions

All authors listed have made a substantial, direct and intellectual contribution to the work, and approved it for publication.

### Conflict of Interest Statement

The authors declare that the research was conducted in the absence of any commercial or financial relationships that could be construed as a potential conflict of interest.
